# Biofortified Beverage with Chlorogenic Acid from Stressed Carrots: Anti-Obesogenic, Antioxidant, and Anti-Inflammatory Properties

**DOI:** 10.3390/foods12213959

**Published:** 2023-10-30

**Authors:** Alejandro Gastélum-Estrada, Griselda Rabadán-Chávez, Edwin E. Reza-Zaldívar, Jessica L. de la Cruz-López, Sara A. Fuentes-Palma, Luis Mojica, Rocío I. Díaz de la Garza, Daniel A. Jacobo-Velázquez

**Affiliations:** 1 Tecnológico de Monterrey, Escuela de Ingeniería y Ciencias, Campus Guadalajara, Av. General Ramón Corona 2514, Zapopan 45201, Jalisco, Mexico; a01229318@tec.mx (A.G.-E.); lisset1395@hotmail.es (J.L.d.l.C.-L.);; 2 Tecnológico de Monterrey, Institute for Obesity Research, Av. Eugenio Garza Sada 2501 Sur, Monterrey 64849, Nuevo León, Mexicorociodiaz@tec.mx (R.I.D.d.l.G.); 3 Food Technology, Centro de Investigación y Asistencia en Tecnología y Diseño del Estado de Jalisco, Unidad Zapopan, Camino Arenero 1227, El Bajío del Arenal, Zapopan 45019, Jalisco, Mexico; lmojica@ciatej.mx; 4 Tecnológico de Monterrey, Escuela de Ingeniería y Ciencias, Campus Monterrey, Av. Eugenio Garza Sada 2501 Sur, Monterrey 64849, Nuevo León, Mexico

**Keywords:** nutraceutical carrot juice, stressed carrots, wounding stress, antioxidant juices, anti-inflammatory juice, chlorogenic acid, anti-obesogenic

## Abstract

Using wounding stress to increase the bioactive phenolic content in fruits and vegetables offers a promising strategy to enhance their health benefits. When wounded, such phenolics accumulate in plants and can provide antioxidant, anti-inflammatory, and anti-obesogenic properties. This study investigates the potential of using wounding stress-treated carrots biofortified with phenolic compounds as a raw material to extract carrot juice with increased nutraceutical properties. Fresh carrots were subjected to wounding stress via slicing and then stored at 15 °C for 48 h to allow phenolic accumulation. These phenolic-enriched slices were blanched, juiced, and blended with orange juice (75:25 ratio) and 15% (*w*/*v*) broccoli sprouts before pasteurization. The pasteurized juice was characterized by its physicochemical attributes and bioactive compound content over 28 days of storage at 4 °C. Additionally, its antioxidant, anti-inflammatory, and anti-obesogenic potentials were assessed using in vitro assays, both pre- and post-storage. The results reveal that juice derived from stressed carrots (SJ) possessed 49%, 83%, and 168% elevated levels of total phenolics, chlorogenic acid, and glucosinolates, respectively, compared to the control juice (CJ) (*p* < 0.05). Both juices reduced lipid accumulation in 3T3-L1 cells and nitric oxide production in Raw 264.7 cells, without significant differences between them. SJ further displayed a 26.4% increase in cellular antioxidant activity. The juice’s bioactive characteristics remained stable throughout storage time. In conclusion, the utilization of juice obtained from stressed carrots in a blend with orange juice and broccoli sprouts offers a promising method to produce a beverage enriched in bioactive compounds and antioxidant potential.

## 1. Introduction

Obesity is a leading public health concern worldwide because it is a major risk for developing chronic, non-communicable diseases such as diabetes, dyslipidemia, hypertension, cardiovascular disease, and cancer. It has been on the rise for the past 30 years, and the shift from fresh and unprocessed foods to ultra-processed products high in sugar, salt, and fat is considered among the main drivers [1]. Eating fresh vegetables is encouraged by healthy dietary traditions, and it is among the main lifestyle interventions to reduce weight gain. Moreover, solid evidence shows that they are the main source of phytochemicals with the potential to counteract obesity by modulating adipose tissue metabolism [2].

Carrot (*Daucus carota*) is one of the most popular root vegetables grown throughout the world. It is a good source of some minerals (Ca, P, Fe, K, and Mg), vitamins (B1, B2, B3, folic acid, and ascorbic acid) and dietary fiber (cellulose > hemicellulose > pectin > lignin), and it has low calorific value and high health benefits, including prevention of constipation, regulation of blood sugar, and prevention of certain forms of cancers [3]. Carrots are also a significant source of carotenoids (β-carotene, α-carotene, and lutein) that, apart from being the main precursors of vitamin A, have been reported to have potent in vivo antioxidant, anticarcinogenic, antimutagenic, immune-enhancing, and photoprotective properties that decrease the risk of developing degenerative diseases such as cancer, cardiovascular disease, age-related macular degeneration, and cataract formation [4,5,6,7].

In addition to their nutritional value, carrots are considered a functional food due to their appreciable amounts of health-promoting phytochemicals. Hydroxycinnamic acids and their derivatives are the main classes of phenolics in carrots [8]. Chlorogenic acid (CHA) is the major hydroxycinnamic acid, representing 42.2–61.8% of total phenolic compounds in different carrot tissues. In vivo studies in animal models and humans have demonstrated that CHA can act as a preventive and/or therapeutic agent against metabolic syndrome due to its antioxidant, anti-inflammatory, anti-obesity, hypolipidemic, hypoglycemic, and antihypertensive effects [9].

It has been demonstrated that the application of controlled abiotic stresses (i.e., wounding, water loss, UV light, etc.) to carrots after harvest increases the synthesis of secondary metabolites with health-promoting properties [10,11]. Wounding stress (i.e., cutting, shredding) is an easy-to-apply method that has been shown to increase the accumulation of phenolic compounds, mainly CHA, in carrots [12].

Global sugar consumption keeps exceeding the WHO’s sugar guideline of <10% of total calories/day for both children and adults and this overconsumption of sugar is one of the main factors promoting overweight and obesity worldwide. Sugar-sweetened beverages are a major source of added sugars in the diet. Moreover, a robust body of evidence has linked their habitual intake with weight gain and a higher risk of type 2 diabetes mellitus, cardiovascular diseases, non-alcoholic fatty liver disease, and some cancers [13]. As public health measures continue to call for reductions in the intake of sugar-sweetened beverages to prevent weight gain and cardiometabolic diseases, there is a growing interest in developing healthier, natural, functional, and low-sugar beverages [14]. This could be the case with plant-based juices from vegetables and fruit, containing vitamins, minerals, dietary fiber, phytochemicals, and fewer calories and sugar.

Thus, the aim of the present work was to produce a chlorogenic acid-rich juice using carrots stressed with wounding mixed with broccoli (*Brassica oleracea* var. *italica*) sprouts and orange (*Citrus sinensis*) juice, and evaluate its shelf-life stability and in vitro antioxidant, anti-inflammatory, and anti-obesogenic potential. By adding orange juice to the blend, we expected to increase vitamin C and carotene content. On the other hand, we added broccoli sprouts as a rich source of dietary fiber and glucosinolates that turn into isothiocyanates after the plant material is damaged, chewed, or blended. The latter has been reported as a highly anti-cancer and anti-inflammatory phytonutrient [15,16].

## 2. Materials and Methods

### 2.1. Reagents and Plant Material

Carrots, oranges, and broccoli sprouts were purchased at a local supermarket (Walmart, Zapopan, Jalisco). HPLC grade water was obtained from a Mili Q Ultrapure water system (Merck Millipore, Billerica, MA, USA). Solvents (HPLC and reagent grades) including methanol, methyl tert-butyl ether (MTBE), acetonitrile, acetone, and ethanol; reagents for colorimetric and chromatography assays including phosphate buffer, dithiothreitol, N-Ethylmaleimide, trichloroacetic acid (TCA), phosphoric acid, α-α′-bipyridil, ferric chloride, Sephadex A25, and sodium acetate; chemical standards, including ascorbic acid, chlorogenic acid, ferulic acid, p-coumaric acid, β-carotene, desulfoglucoraphanin, sinigrin, and quercetin, and microbiological mediums including nutritive agar, violet red bile agar and potato dextrose agar, were purchased from CTR Scientific (Zapopan, Jalisco, México).

Reagents used for in vitro assays including Dulbecco’s modified eagle medium (DMEM), DMEM High Glucose, fetal bovine serum (FBS), streptomycin, newborn calf serum, (3-(4,5-dimethylthiazol-2-yl)-2,5-diphenyltetrazolium bromide) (MTT), lipopolysaccharides (LPS), Griess Reagent System, dichlorodihydrofluorescein diacetate (DCFH-DA), 2,2′-azobis (2-amidinopropane) dihydrochloride (AAPH), Hank’s Balanced Solution, isobutyl-methyl-xanthin, dexamethasone, insulin, Oil Red O (ORO), isopropanol, paraformaldehyde (PFA), and phosphate-buffered saline (PBS) were also purchased from CTR Scientific (Zapopan, Jalisco, México).

### 2.2. Juice Preparation and Storage Studies

Carrots, broccoli sprouts, and oranges were sanitized with sodium hypochlorite (200 ppm, pH 6.5) for 5 min, and excess water was removed before processing. Wounding stress was applied to half of the carrots 48 h before juice preparation by slicing and incubating at 15 °C, according to Santana-Gálvez et al. [17]. Stressed (slices stored for 48 h at 15 °C) and non-stressed (sliced without storage) carrots were blanched separately (100 g of carrots per 1 L water) at 80 °C for 6 min and ground in a food processor. The carrot juice was extracted by pressing ground carrots through two layers of sterile cheesecloth.

Orange juice was extracted using a manual lever juicer and poured through a strainer to remove pulp and seeds. Carrot and orange juice were mixed in a 75/25% (*v*/*v*) proportion. Broccoli sprouts were added in a 15% (*w*/*v*) ratio and blended in a food processor (FP4200B, Black + Decker, Middleton, WI, USA) until a homogeneous mixture was obtained.

**Figure 1:**
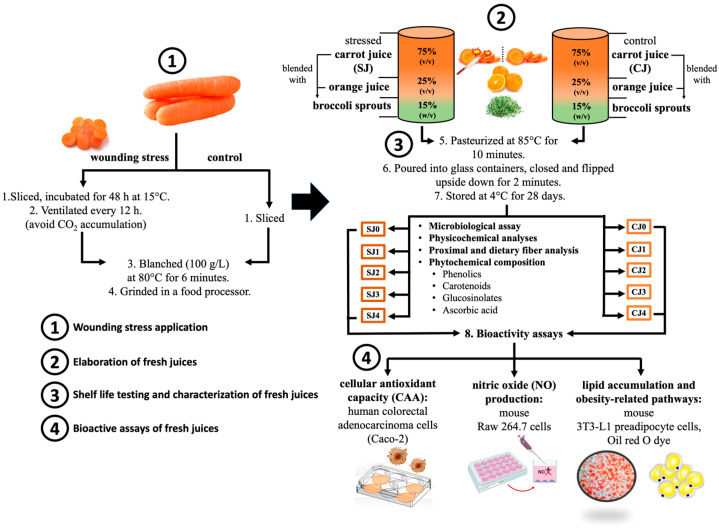
Schematic diagram of experimental set-up for chlorogenic acid-rich juice preparation and evaluation of its acceptability, shelf-life stability, and in vitro antioxidant, anti-inflammatory, and anti-obesogenic potential.

The mixture of juices containing carrot juice obtained from non-stressed carrots is further referred to as control juice (CJ), while the juice prepared from stressed carrots is further referred to as stressed juice (SJ). The CJ and SJ were pasteurized at 85 °C for 10 min and hot-filled into glass containers (Ball^®^ Mason Jars, 500 mL). The containers were cooled at room temperature and stored under refrigeration (at 4 ± 1 °C) for 28 days. Samples were taken every 7 days for microbiological, physicochemical, and phytochemical evaluations. Moreover, for each sampling time, a portion of the juice was freeze-dried (FreeZone 2.5 Liter Benchtop Freeze Dryer, Labconco, Kansas City, MO, USA) to stabilize the sample before further analyses. Figure 1 summarizes the experimental setup for obtaining the juices and the subsequent physicochemical, microbial, and biological in vitro evaluations.

### 2.3. Microbiological Validation

Mesophilic factors, total coliforms, fungi, and mold presence were determined on CJ and SJ samples according to the Mexican Official Standards NOM-092-SSA1-1994, NOM-210-SSA1-1994, and NOM-111-SSA1-1994 [18,19,20], weekly for 28 days to assess the microbial stability of the juice. Nutritive agar was used for mesophilic aerobic count determination by surface inoculation and incubation for 48 h at 37 °C. Violet Red Bile Agar was used for total coliform quantification by the pour plate method and incubation for 24 h at 35 °C. Finally, mold and yeast counts were determined using potato dextrose agar at pH 3.5 (adjusted with 10% formic acid) by surface inoculation and incubation for 7 d at 30 °C. Both juices presented <10 CFU/mL for all microorganisms tested; thus, they were microbiologically stable after pasteurization and during the evaluated storage period.

### 2.4. Physicochemical Analyses

Total soluble solids (°Brix), insoluble solids, titratable acidity, pH, and color were measured at 0, 14, and 28 d at room temperature in CJ and SJ samples. An Orion Star A211 pH meter (Thermo Scientific, Waltham, MA, USA) and a hand refractometer (PAL-UREA, Atago, Tokyo, Japan) were used to determine the pH and °Brix, respectively, according to manufacturer instructions. Insoluble solids were determined according to the NMX-F-180-1986 method, while titratable acidity was analyzed by the AOAC 942.15 method. Colorimetric analysis in the CIELAB scale (L*: brightness; a*: redness; b*: yellowness) was performed using a benchtop colorimeter (CR-5, Konica Minolta Holdings Ltd., Tokyo, Japan).

### 2.5. Proximal and Dietary Fiber Analysis

The proximate compositions of the juice samples were expressed on a wet-weight basis and were determined according to Official Mexican Regulations (NMX). Moisture content was determined by drying in a hot air oven at 100 ± 5 °C to a constant weight [21]. Crude protein content was determined by the Kjedahl method [22]. Crude fat content was determined by extraction with petroleum ether using a Soxhlet system [23]. After the crude fat analysis, the samples were used to investigate the crude fiber content by sequentially extracting the sample with 1.25% H_2_SO_4_ and then 1.25% NaOH. After the digestion, the samples were dried, and the weight of each dried sample was recorded [24]. Samples were then used to determine the ash content by incineration at 550 ± 5 °C in the presence of oxidizing agents [25]. The carbohydrate content was calculated from the sum of the percentages of crude protein, ash, fat, and crude fiber subtracted from 100. Insoluble, soluble, and total dietary fiber were determined by the enzymatic–gravimetric method [26].

### 2.6. Extraction, Identification, and Quantification of Phytochemicals

#### 2.6.1. Phenolics

Phenolic compounds were determined according to Santana-Gálvez et al. [17]. Briefly, the juice was centrifuged (3134× *g*, 4 °C, 30 min), and the supernatant was filtered using a nylon syringe filter (0.22 μm) and injected into a UHPLC-PDA system (ACQUITY Arc, Waters, Milford, MA, USA). Compounds were separated at 40 °C using a Waters Cortecs C18 column (4.6 × 150 mm, 2.7 μm pore size). The mobile phases were (A) Mili-Q water adjusted to pH 2.4 using orthophosphoric acid and (B) methanol. Gradient elution was 0/90, 13.2/65, 15.6/2 and 19.2/90 (min, % phase A) at 1 mL/min flow. The injection volume was 20 μL. Individual phenolic compounds, including CHA, ferulic, and p-coumaric acid, were detected at 320 nm and quantified using specific standard curves. Total phenolics (mg/L) were determined as the sum of all identified compounds and reported as CHA equivalents.

#### 2.6.2. Carotenoids

Carotenoids were analyzed as indicated by Santana-Gálvez et al. [17] with slight modifications. The extraction from 0.2 g of freeze-dried samples was done using 2 mL of acetone under dark conditions. The mixture was vortexed and this allowed extraction for 15 min before centrifugation (3134× *g*, 4 °C, 15 min). The supernatant was filtered using a nylon syringe filter (0.22 μm). A methanol/MTBE/water (50/45/5) isocratic system (15 min total run) was used as the mobile phase in the same UHPLC-PDA system used for phenolics determination. A total of 20 μL of the sample was injected and separated with a Waters Cortecs C18 column. The detection of carotenoids was done at 450 nm and they were quantified using a β-carotene standard curve.

#### 2.6.3. Glucosinolates

Glucosinolates were extracted, desulfated, and quantified as desulfoglucosinolates, according to Villarreal-García et al. [27]. For extraction, 0.2 g of freeze-dried juice was added to 4 mL of 70% ethanol/water (*v*/*v*) solution at 65 °C and vortexed. The mixture was cooled and centrifuged (3134× *g*, 15 min, 4 °C), and the supernatant recovered. Glucosinolates were desulfated using disposable polypropylene columns previously prepared with Sephadex A-25 in sodium acetate (8.7% *w*/*v*). Sample supernatant (3 mL) was added to the prepared column with sinigrin as the internal standard (50 μL, 3 mM) and washed twice with 500 μL of water and sodium acetate. Sulphatase (75 μL) was added and incubated for 12 h at room temperature. Desulfoglucosinolates were eluted after incubation using 1.25 mL of water. The eluted solution (20 μL) was injected into the UHPLC-PDA Acquity Arc system and separated using a Waters Cortecs C18 column. Water (A) and acetonitrile (B) were used as mobile phases using gradients 0/100, 17/20, 18/0 and 19.2/0 (min, % phase A) at a constant flow of 1.5 mL/min. Total glucosinolates were quantified as desulfoglucoraphanin equivalents using a standard calibration curve at 227 nm.

#### 2.6.4. Reduced, Oxidized, and Total Ascorbic Acid

Reduced, oxidized, and total ascorbate contents were measured according to Gillespie and Ainsworth [28]. Briefly, 100 μL of phosphate buffer (75 mM) and 200 μL of supernatant of previously centrifuged juice (3134× *g*, 15 min) were added in duplicate in 2 mL tubes. In one tube, 100 μL of DTT (10 mM) was added to reduce ascorbate anion and incubated (10 min). Then, 100 μL NEM (0.5%) was further added to stop the reaction. Water was added to the other tube for reduced ascorbic acid measurement. For both tubes, 500 μL TCA (10%), 400 μL phosphoric acid (43%), 400 μL α-α’-bipyridyl (4%), and 200 μL FeCl3 (3%) were added and incubated at 37 °C for 1 h. Each tub’s solution (200 μL) was transferred to a 96-well microplate, and absorbance was measured at 525 nm using a microplate reader (Varioskan Lux, TermoFisher, Vantaa, Finland). Absorbance readings were compared to the standard curves for the quantification of reduced ascorbic acid (AA), and oxidized species were calculated as the difference between total and reduced AA in the sample.

### 2.7. Cell Culture

Mouse macrophage (Raw 264.7), human colorectal adenocarcinoma (Caco-2), and mouse embryonic fibroblast (3T3-L1) were purchased from the American Type Culture Collection (ATCC, Manassas, VA, USA) and used to evaluate the anti-inflammatory potential, cellular antioxidant activity, and lipid metabolism evaluation, respectively. Raw 264.7 and Caco-2 cells were maintained in DMEM/F12 supplemented with 10% FBS and 1% antibiotic (10,000 units penicillin and 10 mg streptomycin/mL), while the 3T3-L1 was maintained in DMEM high glucose supplemented with 10% calf serum and 1% antibiotic at 37 °C and 5% CO_2_.

#### 2.7.1. MMT Assay

The cytotoxicity of CJ and SJ was evaluated in all cell lines using MTT reagent [29]. Briefly, different treatment dilutions were applied to each cell culture and incubated for 24 h, then the medium was replaced with a MTT solution (5 mg/mL) and incubated for 2 h. Absorbance was measured at 570 nm, and cell viability was expressed as a percentage compared to the non-treated control.

#### 2.7.2. Evaluation of Anti-Inflammatory Potential

Anti-inflammatory activity was analyzed using Raw 264.7 cells stimulated by LPS [30]. In inflammatory conditions such as LPS stimulation, the TLR-4 and the proinflammatory cytokines induce nitric oxide production, which is converted to peroxynitrite and nitrite [31]. The Griess reaction is a method of analysis of nitrite (one of two primary stable and nonvolatile breakdown products of nitric oxide) based on the nitrite reaction with sulfanilamide under acidic (phosphoric acid) conditions to form a diazonium cation, which subsequently couples to N-1-naphthyl ethylenediamine dihydrochloride to produce a red–violet-colored water-soluble azo dye [32]. Briefly, cells were seeded in a 96-well plate and incubated for 24 h. Cells were incubated for 1 h with 5% CJ and SJ. Then, treatments were replaced with LPS (2.5 μg/mL) and incubated for 24 h. Nitrite production was measured with the Griess Reagent System (Promega, G2930) according to the manufacturer’s instructions. Anti-inflammatory potential was reported as nitrite oxide production inhibition of treated cells compared to a non-treated control.

#### 2.7.3. Cellular Antioxidant Activity (CAA)

The level of reactive oxygen species (ROS) produced by cells in the presence of treatments was determined using Caco-2 cells [33]. Briefly, cells were cultivated in a black 96-well plate at 3 × 10^4^ cell density. A solution of DCFH-DA (60 μM) and 5% juice treatments in DMEM medium was added to the plate, incubated for 1 h, and then washed twice using PBS. Afterward, a 500 μM AAPH solution in Hank’s Balanced Salt Solution was added to the plate. Fluorescence excitation (485 nm) and emission (538 nm) were immediately measured every 5 min for 1 h at 37 °C (13 readings were performed in total). The effectiveness of antioxidant treatments for different treatments was quantified by examining the percent reduction in the fluorescence of treated cells compared to a non-treated control, calculated as follows:

$$\mathrm{CAA}\;\mathrm{unit}=\%\;\mathrm{reduction}=1-\frac{AUC\;sample}{AUC\;control\;}\;*\;100$$

CAA units were reported as mean ± standard error.

#### 2.7.4. Evaluation of Antiadipogenic Potential

##### 3T3-L1 Differentiation and ORO Staining

Total lipid accumulation, including the ORO staining, free glycerol, and triglyceride accumulation, was determined after the 3T3-L1 cell differentiation. Cells were seeded in a 24-well plate with DMEM supplemented with 10% newborn calf serum. Adipocyte differentiation was induced with DMEM high glucose supplemented with 10% FBS, 0.5 mM isobutyl-methyl-xanthin, 0.25 μM dexamethasone, and 5 μg/mL of insulin [34]. After three days, differentiation media was replaced with DMEM high glucose supplemented with 10% FBS and 5 μg/mL of insulin. On day 7, the medium was changed to DMEM high glucose containing 10% FBS. Treatments were added every time the medium changed. ORO staining was performed until the 12th differentiation day [34]. An ORO stock solution was prepared by stirring 0.5% Oil Red O in isopropanol overnight and filtered through a 0.2 μm filter. Fresh ORO working solution was prepared by mixing stock solution with distilled water (6:4). Cells were washed twice with PBS 0.01 M (pH 7.4), and fixed with PFA 4% for 15 min. Cells were washed again with PBS and then with isopropanol 60%. The previously prepared ORO solution was added for 30 min, and cells were washed again with PBS. Cells were observed by microscope (OPTIKA IM-3, OPTIKA, Italy) and documented by assembled camera (Optikam PRO8 Digital Camera C-P8, OPTIKA, Italy) using the manufacturer’s software (OPTIKA Pro-View, OPTIKA, Italy). Finally, the stained lipid droplets were incubated with isopropanol 60 to extract the dye and then measured by absorbance at 490 nm. Data were reported as a percentage against non-treated control absorbance.

##### Free Glycerol Assay

Free glycerol was determined after differentiation using a colorimetric kit (Abcam, Cambridge, UK) following the manufacturer’s instructions. Briefly, 25 μL of cell culture supernatant and 100 μL of Free Glycerol Assay Reagent were added to a 96-well plate, and incubated for 15 min before reading absorbance at 540 nm. The glycerol content was determined using a glycerol standard curve (0–125 μg/mL). The results are reported as a percentage against the non-treated control.

##### Triglyceride Enzymatic Assay

Intracellular triglyceride accumulation was determined using a lipase-based kit (Abcam, Cambridge, UK) according to the manufacturer’s instructions. Briefly, the harvested cells were resuspended in 1 mL of 5% Triton X-100 solution, heated to 90 °C in a water bath for 5 min, then cooled to room temperature. This step was repeated twice. The solution was centrifuged at 10,000× *g* for 2 min, and the supernatant was transferred to a new tube and diluted 10-fold. Triglyceride quantification was set up with 50 µL of supernatant samples. Then, 2 µL of cholesterol esterase/lipase solution was added and incubated for 20 min at RT. Finally, 50 µL of triglyceride reaction mix was added and incubated for 60 min under dark conditions. Triglycerides were quantified by absorbance at 570 nm and are reported as a percentage against the non-treated control.

### 2.8. Statistical Analysis

Three independent experiments were performed (*n* = 3). Statistical analyses were performed using the mean value of samples and their standard error. Analyses of variance (ANOVA) were conducted to determine the main effects and interactions and mean separation was performed using the LSD test (*p* < 0.05). Statistical analyses were conducted with the JMP software version 17.0 (SAS Institute Inc., Cary, NC, USA).

## 3. Results and Discussion

### 3.1. Proximate and Dietary Fiber Composition

The proximate compositions of control (CJ) and stressed (SJ) juices are shown in Table 1. There were no significant differences in moisture, ash, total carbohydrates, crude fiber, total dietary fiber, and soluble dietary fiber between CJ and SJ samples. Total fat and insoluble dietary fiber contents were higher in CJ compared to SJ (28.5 and 7.4%, respectively), whereas protein content was 1.06% higher in SJ compared to CJ.

Both juices showed a high moisture content (above 90%), like the raw materials they were made of (carrots, oranges, and broccoli sprouts). Likewise, these results are comparable to moisture content values (86.04–91%) previously reported for different fruit and vegetable juices [35,36,37,38].

It has been previously reported that juice produced from carrots treated with wounding stress showed increased ash content as a function of higher amounts of sodium, calcium and potassium [17]. However, this study showed no significant differences in ash content between CJ and SJ samples. Still, ash content falls within the range 0.38–0.58% FW reported in juices from raw carrots and citrus fruits [39,40,41], and is higher than the range (0.016–0.219%) reported in processed fruit juices [42]. The addition of broccoli sprouts to both CJ and SJ (15% *w*/*v*) may also be contributing to their ash content, since they are rich sources of minerals such as K, Ca, Mg, P and Fe [43,44].

The lipid and protein contents of both juice samples were very low, which is common for fruits and vegetables. Given that carrot juice accounts for the major proportion of both juices (75% *v*/*v*), the protein content is in accordance with the values reported in raw carrots (0.7–0.9%) [3]. The contribution of broccoli sprouts may also be taken into consideration, since they have higher protein contents than carrots (~4.7%) [43]. Additionally, the crude protein content falls within the range reported in juices from raw carrots (0.6–1.75%) [37,38,41]. Differences could be due to variations in maturity levels, varieties, and geographical locations.

Commercially made juices often contain little or no fiber, compared to whole fruits and/or vegetables [42]. In this study, total dietary fiber content can be mainly attributed to the addition of broccoli sprouts since carrot pomace was discarded after juice extraction, and pulp was separated from the orange juice before mixing it with carrot juice.

Lignin accounts for ~32% of the dietary fiber content of carrot pomace (on dry weight basis), and wounding stress in carrots has been reported to significantly increase its content, secondary to a higher accumulation of phenolics. As an indigestible polysaccharide, lignin can have a lipid-sequestrating effect (before juice extraction) that may explain the lower fat content in SJ, compared to CJ (<0.10% vs. 0.14% FW, respectively) [45].

### 3.2. Physicochemical Characterization and Changes during Storage

Table 2 shows changes in physicochemical parameters (pH, acidity, °Brix, insoluble solids, and color) of CJ and SJ determined at three times points (0, 14, and 28 days) of the storage study (storage at 4 °C). Before storage (T_0_), the pH and total soluble solids values were lower in SJ than in CJ. Conversely, insoluble solids were higher in SJ than in CJ. The pH values of both CJ and SJ decreased after 28 days of storage. The decline in the pH of SJ was accompanied by an increase in acidity value, whereas the acidity of CJ remained stable. Total soluble solids showed a significant decrease in CJ and SJ samples at the end of the shelf-life study (7.9 and 3.4%, respectively, compared to T_0_), with CJ being the sample with the lowest values. Insoluble solids showed a slight decrease in both CJ and SJ by the end of the shelf-life study.

The initial pH values of both CJ and SJ (4.9 and 4.8, respectively) fell between the pH reported for orange (3.3–3.7) [46,47,48] and carrot juices (5.9–6) [17,49,50]. Despite the slight decline in pH during the storage period, pasteurization and orange juice addition to the blend contributed to maintain more stable pH and tritable acidity values and to extend the shelf-life of CJ and SJ during the 28-day period at 4 °C [49]. Moreover, it has been reported that increased chlorogenic acid derivative contents (secondary to wounding stress) is correlated with a lower pH and a higher acidity [17]. Non-enzymatic reactions of sugars, amino acids and ascorbic acid are well-known phenomena that take place during the storage of citrus juices [51,52]. Thus, the significant decrease in soluble solids in CJ and SJ during storage may result from Maillard reaction development after pasteurization. The addition of broccoli sprouts to the blend is the main reason for the high insoluble solids contents in both CJ and SJ (15% *w*/*v*). The latter is consistent with dietary fiber content in both experimental juices.

Color parameters (L*, b*, Chroma and Hue) were significantly higher in fresh SJ (T_0_) compared to CJ (Table 3). However, at the end of the storage period (T_28_), the initial values of SJ showed a significant decrease. Conversely, the color parameters of CJ samples showed a significant increase at T_28_. The decreased brightness (L*) of both CJ and SJ, compared to values reported for carrot (24.9–54.6) [53,54,55] and orange fresh juices (45–58.2) [56,57,58], could be a result of blending broccoli sprouts with carrot and orange juice. Interestingly, after the storage period, the L* value of CJ significantly increased (1.8-fold, *p* < 0.05) compared to its initial value. Compared to CJ, lower a* values of SJ at T_28_ may be related to its lower carotenoid content. Lower yellowness (b*) values of CJ and SJ, compared to values reported for carrot (35.7–41.2) and orange juices (43.6–51), may also be related to the addition of broccoli sprouts to the blend. The darkening of carrot shreds during wounding stress incubation may affect the a* and b* values of SJ during storage period [17].

### 3.3. Phytochemical Composition and Changes during Storage

#### 3.3.1. Phenolics

**Figure 2:**
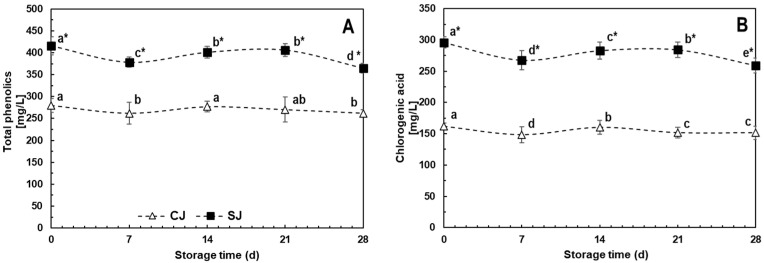
Total phenolic (**A**) and chlorogenic acid (**B**) content during storage (28 days, 4 °C) of juices prepared with either non-stressed (CJ) or stressed carrots (SJ) as an ingredient. Values represent the means of three replicates with their standard error bars. Different letters represent statistical differences for the same treatment at different storage times. Values with an asterisk (*) indicate statistical differences between treatments at a specific storage time. Statistical differences were determined by the LSD test (*p* < 0.05).

The phenolic contents for SJ and CJ at T_0_ were 416.3 and 280.1 mg/L, respectively, while the CHA concentrations were 296.0 and 162.2 mg/L (Figure 2), representing around 71% and 56% of total phenolic compounds detected in the juice. Other compounds identified in minor concentrations, which quantities did not change due to wounding stress application in carrots raw material, included ferulic and p-coumaric acids. The increases in phenolics and CHA due to the use of stressed (SJ) compared to un-stressed (CJ) carrots for juice preparation were 49% and 83% at T_0_. This difference can be attributed to the application of wounding stress before juice production. Regarding stability during storage, CJ showed a 6% degradation for both total phenolics and CHA during 28-day storage at 4 °C, while SJ showed a slightly higher degradation rate of 12% for both quantifications (Figure 2A,B). A higher amount of total phenolics (39.3%) and CHA (70.8%) was detected in SJ compared to CJ at the end of the storage period.

A previous study evaluated the effects of wounding stress prior to juice extraction on the content of phenolics. The authors have reported that the juice obtained from stressed carrots showed 174 and 290% higher phenolics and CHA after pasteurization as compared with the juice obtained from non-stressed carrots [17]. The reduced accumulation observed in this study could be attributed to differences in the initial levels of phenolic compounds present in the raw material, before the application of the stress, and also to the interaction with orange juice and broccoli sprouts, ingredients not used in the previous study.

The slight degradation rates observed during storage, on the other hand, fit with the report of Ianni and others [59], who studied the stability of chlorogenic acid in aqueous solutions and observed that the compound was highly stable for up to 30 days in pure water even after thermal (microwave) treatment. The stability of CHA, being the most highly elicited compound by wounding stress, has beneficial implications for the final product formulation, given its longer shelf-life without the loss of its potential effects.

#### 3.3.2. Carotenoids

**Figure 3:**
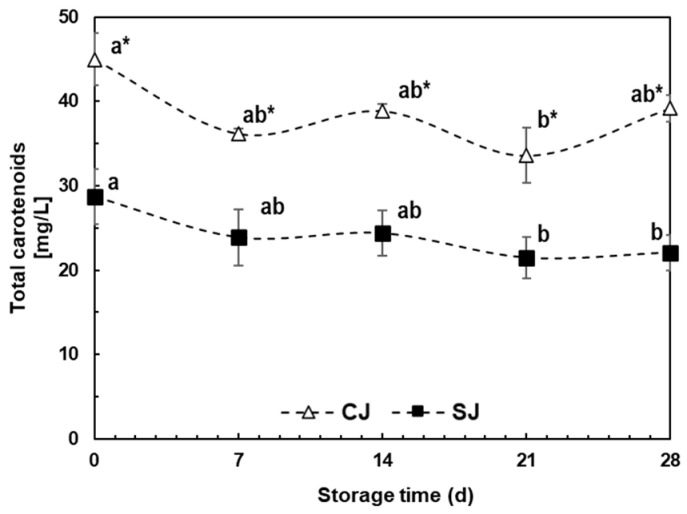
Total carotenoid content during storage (28 days, 4 °C) of juices prepared with either non-stressed (CJ) or stressed carrots (SJ) as an ingredient. Values represent the mean of three replicates with their standard error bars. Different letters represent statistical differences for the same treatment at different storage times. Values with an asterisk (*) indicate statistical differences between treatments at a specific storage time. Statistical differences were determined by the LSD test (*p* < 0.05).

The total carotenoid content was 36.2% lower for SJ than CJ at T_0_ (Figure 3). This difference remained during storage time, varying between 33.9 and 43.6%, and the maximum difference was observed at day 28 of storage (T_28_). The accumulated degradation during storage for CJ and SJ were 13 and 23%, respectively.

Carrot is one of the most essential sources of β- and α- carotene; orange is considered a rich source of xanthophylls, while broccoli sprouts contain small portions of lutein; these are proposed as the main components in this beverage [60]. Previous studies have mentioned that wounding stress and 48 h incubation did not affect the carotenoid content in carrots or the produced juice [17,61]; the reduction in this study may be attributed to the method of processing carrot slices after blanching. Carrot slices were directly ground using a food processor with a clear surface instead of cooling them down and then extracting with a juice extractor (extraction under dark conditions); this difference could lead to the higher thermal and light degradation at the juice extraction moment, giving the known lability of carotenoids [62].

Chen et al. [63] reported this low degradation rate of carotenoids in carrot juice under the same conditions (dark storage, 4 °C). The slight acidity in orange juice (pH 4.7–4.9) may also lead to preserving carotenoid content, as reported by Bell et al. [64], who noted that acidic conditions may help to reduce degradation while basic conditions accelerate it.

#### 3.3.3. Ascorbic Acid

**Figure 4:**
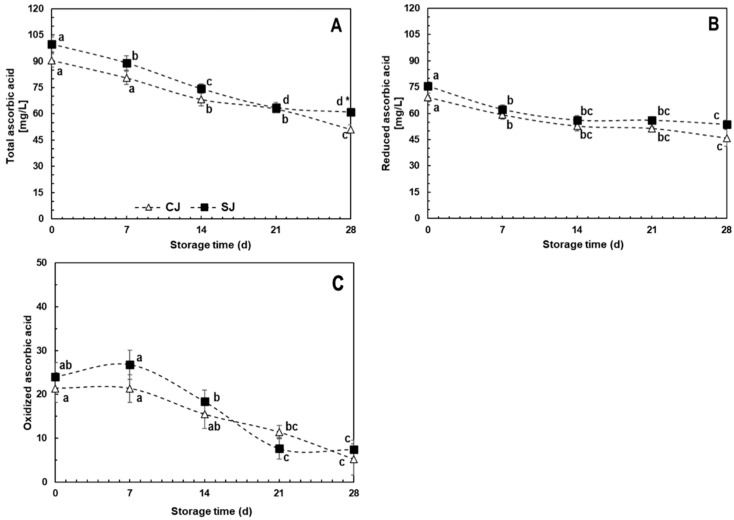
Total (**A**), reduced (**B**), and oxidized (**C**) ascorbic acid content during storage (28 days, 4 °C) of juices prepared with either control (CJ) or stressed carrots (SJ) as an ingredient. Values represent the mean of three replicates with their standard error bars. Different letters represent statistical differences for the same treatment at different storage times. Values with an asterisk (*) indicate statistical differences between treatments at a specific storage time. Statistical differences were determined by the LSD test (*p* < 0.05).

The total AA contents in fresh juice (T_0_) were 99.7 and 90.5 mg/L for SJ and CJ, respectively, with no significant difference between the treatments (Figure 4). The same behavior was observed in reduced and oxidized AA quantifications (Figure 4B,C), where the average contents were 72.4 and 22.7 mg/L, respectively. No statistical difference was observed during storage between treatments except at T_28_ when the total AA content in SJ was ≈ 20% higher than CJ (Figure 4A). Likewise, no significant differences were determined for oxidized (Figure 4B) and reduced (Figure 4C) ascorbic acid species.

Of the ingredients present in the beverage, orange juice is considered the most important source of AA given that it can contain up to 500 mg/L, compared to 15.5 mg/L reported in the literature for carrot juice [65,66]. Considering that the beverage herein evaluated contains 25% (*v*/*v*) orange juice, the AA content quantified can be considered appropriate, considering the heath-induced degradation occurring during pasteurization.

In a study performed by Romeo et al. [47], orange juice supplementation with a phenolic concentrate (hydroxytyrosol, 50 ppm) led to a reduction in AA degradation after 30 days at 6 °C; this reduction varied according to the used concentration of phenolics. For this work, the relevant concentration of phenolic compounds in carrot juice may have slowed the AA reduction during storage, mainly in the reduced (active) form, which only decreased 30–33% during storage for both juices, compared to ≈75% of the oxidized form (Figure 4A,B). Increasing AA stability by combinations of ingredient and individual components may increase the product’s commercial value.

#### 3.3.4. Glucosinolates

**Figure 5:**
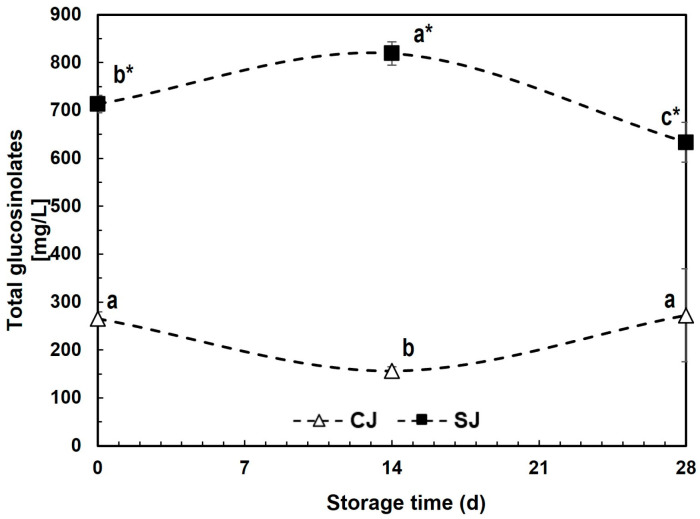
Total glucosinolates content during storage (28 days, 4 °C) of juices prepared with either non-stressed (CJ) or stressed carrots (SJ) as an ingredient. Values represent the mean of three replicates with their standard error bars. Different letters represent statistical differences for the same treatment at different storage times. Values with an asterisk (*) indicate statistical differences between treatments at a specific storage time. Statistical differences were determined by the LSD test (*p* < 0.05).

The total glucosinolates content was 168% higher for SJ than CJ after beverage production (T_0_), with 713.4 and 265.4 mg/L, respectively (Figure 5). During storage, there was no clear degradation rate for glucosinolates, given that for SJ, the reduction was 11.1% when decreasing from 713.4 to 633.7 mg/L, while in CJ, no significant change was observed (Figure 5).

Vegetables of the *Brassicaceae* family, such as broccoli, are considered rich sources of glucosinolates, and the content of this component is even higher in sprouts and makes them a potential source of bioactive compounds for formulation [67]. Naturally, glucosinolates are physically separated from its degradative enzyme myrosinase in the vegetable tissue, but they are liberated upon mechanical damage, infection, or pest attack, allowing them to react and form mainly isothiocyanates [68].

The glucosinolate–myrosinase reaction is considered a complex system affected by different cofactors, such as AA, but also by pH and environmental factors when both elements are released [69]. Glucosinolate content has been reported to be reduced up to 75% when vegetables are shredded or ground within 6 h, mainly by myrosinase activity [70]. The results suggest that SJ has lower myrosinase activity than CJ due to the AA content, lower pH, and higher chlorogenic acid concentration, reducing glucosinolate degradation during blending and thus yielding higher concentrations.

Glucosinolates are considered a stable type of compound, mainly if compared to their derivatives, isothiocyanates, which are known as highly volatile [68]. The slight reduction in glucosinolates observed in SJ could be related to the physicochemical properties of each juice, where factors such as the pH have been reported to affect glucosinolates stability [71].

### 3.4. Antioxidant and Anti-Inflammatory Activity

In order to select the non-cytotoxic concentrations of juices (CJ and SJ at 0 and 28 days of storage), 3T3-L1, Caco-2, and RAW 264.7 cells were exposed for 24 h to different juice concentrations ranging from 1.04 to 6.25% *v*/*v*. Cell viability higher than 85% was achieved in concentrations up to 5% of juice in all cell lines. Therefore, 5% was selected for anti-inflammatory and antioxidant activity, while 1% was used for anti-obesogenic assays, given the longer process needed during the differentiation of 3T3-L1 cells.

**Figure 6:**
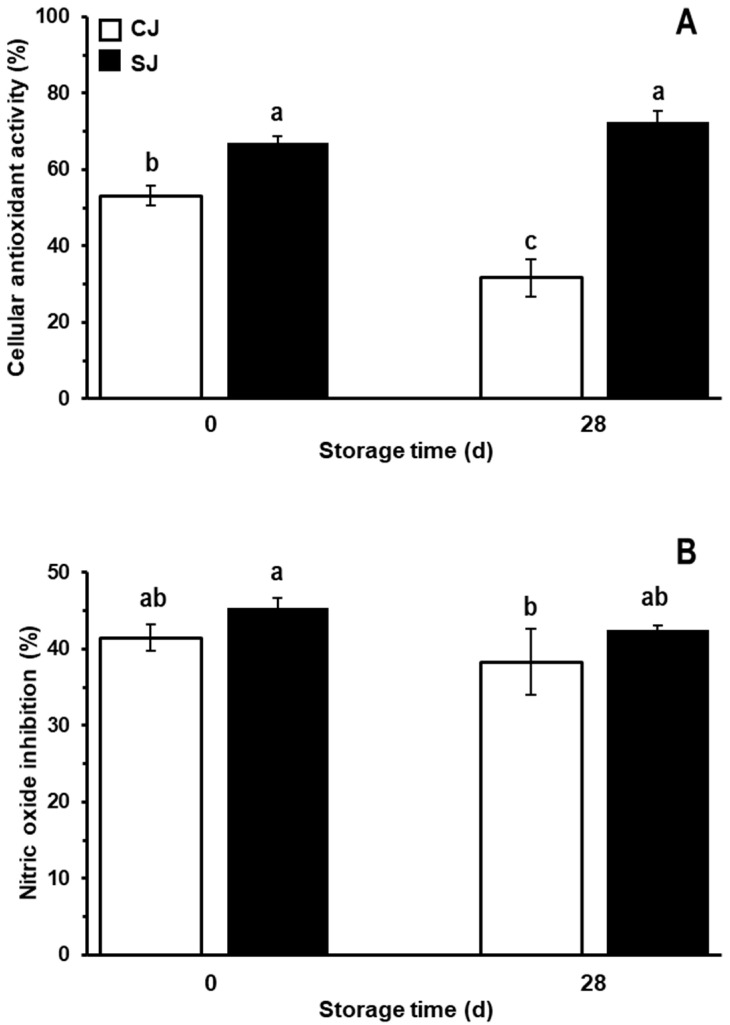
Cellular antioxidant activity (**A**) and nitric oxide cell production inhibition (**B**) of juices prepared with either non-stressed (CJ) or stressed carrots (SJ) as an ingredient at days 0 and 28 of storage at 4 °C. Indicated percentages are a comparison against a non-treated control for each parameter. The control consisted of cells non-treated with any of the juices, and its corresponding cellular antioxidant activity and nitric oxide inhibition would take a reference value of 0%. Values represent the mean of three replicates with their standard error bars. Different letters represent statistical differences for the two treatments at different storage times. Statistical differences were determined by the LSD test (*p* < 0.05).

The cellular antioxidant activity and in vitro anti-inflammatory activity of the juices evaluated are shown in Figure 6. As observed, using carrots treated with wounding stress as raw material for the production of the juice generated a product with 26% higher cellular antioxidant activity (Figure 6A). At T_28_, it was observed that the antioxidant capacity of CJ decreased considerably, by approximately 40% compared to CJ at T_0_. On the other hand, SJ at T_28_ did not present a decrease in this bioactivity compared to SJ at T_0_. Comparing CJ and SJ at T_28_, the SJ presented an antioxidant potential 128.6% higher than CJ.

The antioxidant potential observed for the carrot juices is in accordance with its total phenolic CHA contents (Figure 2B). These data agree with those of other studies denoting that the polyphenolic compounds, abundantly present in fruits, vegetables, cereals, and food-based beverages, are responsible for most of their antioxidant potential [72,73,74,75,76]. Phenolics like CHA exhibit antioxidative properties by chelating metal ions, inhibiting lipid oxidation, inhibiting radical-forming enzymes, and eliminating free radicals. Moreover, CHA can suppress the increase in ROS by activation of the PI3K/AKT signal pathway, protecting cells from oxidative stress by upregulating the FOXO family genes and Bcl-2 [9,75].

On the other hand, as shown in Figure 6B, both juices showed adequate anti-inflammatory activity. There was no statistically significant difference in the production and release of nitrites between the CJ and the SJ at T_0_. Likewise, at T_28_, the juices retained their potential anti-inflammatory effect. Interestingly, although the juice prepared with stressed carrots exhibited higher phenolic content, this higher content did not result in a higher anti-inflammatory effect. This result could be explained in terms of other compounds that could be decreased due to abiotic stress application in the raw material, and which could exert an anti-inflammatory effect but were not quantified in this study. It is essential to point out that the anti-inflammatory and antioxidant potential herein evaluated is the result of the interaction between the compounds present in each juice, which interaction could be additive, antagonistic, or synergistic [77,78]. In this line, the higher concentration of some phenolics, such as CHA, could compensate for the loss of other compounds in SJ, thus maintaining the anti-inflammatory activity observed in CJ [79].

### 3.5. Anti-Obesogenic Potential of Juice Samples

**Figure 7:**
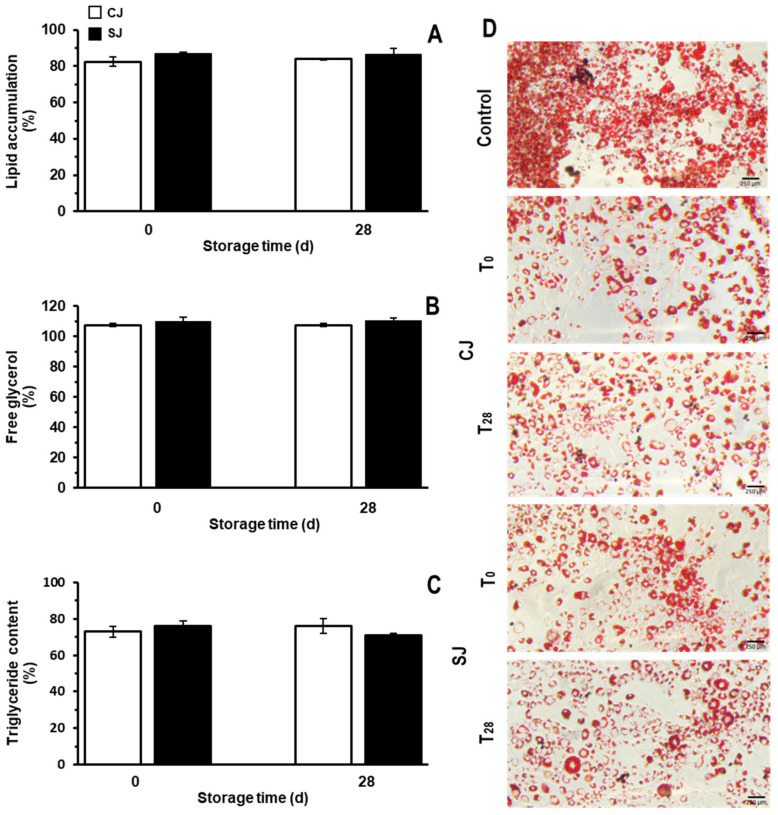
Lipid accumulation (**A**), free glycerol (**B**), triglyceride content (**C**) and Oil Red O staining (**D**) in 3T3-L1 cell culture of juices prepared with either non-stressed (CJ) or stressed carrots (SJ) as an ingredient at days 0 and 28 of storage (T_0_ and T_28_, respectively) at 4 °C. Indicated percentages are a comparison against a non-treated control for each parameter. The control consisted of cells non-treated with any of the juices, and its corresponding lipid accumulation, free glycerol and triglyceride content would take a reference value of 100%. Values represent the means of three replicates with their standard error bars. Different letters represent statistical differences for the two treatments at different storage times. Statistical differences were determined by the LSD test (*p* < 0.05).

The anti-obesogenic potential of the juices was evaluated during the differentiation process of the 3T3-L1 cell line. The effects of the juices on the intracellular accumulation of lipids are shown in Figure 7. At T_0_, the CJ and the SJ decreased the accumulation of lipids by 13.3 and 17.6%, respectively, compared to the control group (Figure 7A). No statistically significant difference in lipid accumulation between the CJ and the SJ at T_0_ was observed. At T_28_, it is observed that the juices maintained the anti-obesogenic effect shown at T_0_. No statistically significant differences between the CJ and the SJ were observed.

Although free-glycerol levels increased after treatment with the juices (Figure 7B), there was no statistical difference between the CJ and SJ. At T_0_, CJ and SJ increased the glycerol levels by approximately 7.6% and 10.2%. At T_28_, the glycerol levels remained practically the same as those at T_0_ (7.5 and 10.8% for CJ and SJ, respectively).

The triglycerides’ quantification coincides with those regarding Oil Red O staining (Figure 7C). At T_0_, there was a reduction in triglyceride levels of 27.1 and 23.8% for the CJ and the SJ, respectively, compared to the control group. No statistically significant differences were observed between the CJ and SJ. At T_28_, the effects of the juices were the same as those observed at T_0_. The quantification of Oil Red O staining is similar to what was observed in Figure 7D; the juices considerably decreased the intracellular Oil Red O staining. The lipid droplets in the control group became larger with a deeper red color, while in the juice-treated cells, this phenomenon decreased. As observed in the anti-inflammatory assay, the application of wounding stress in the raw material did not increase the anti-adipogenic activity of SJ. As described, this could be explained in terms of the modification of the phytochemical profile of carrots exerted by the application of wounding stress [80]. Herein, we only evaluated the phenolics, carotenoids, and glucosinolates. Still, a more detailed metabolomic assay could give us more detail on potential anti-obesogenic compounds that could be modified through wounding stress. Also, the results suggest that although the concentration of phenolics was increased due to wounding stress application in carrots, the concentration in the juice obtained from the stressed tissue was not sufficiently increased to exert an in vitro effect on lipid accumulation [81] and anti-inflammatory potential.

## 4. Conclusions

This study has demonstrated the efficacy of wounding stress in enhancing the phenolic content of carrots, presenting an opportunity to use them as raw materials for producing a juice with health-promoting properties. When these treated carrots were juiced and blended with orange juice and broccoli sprouts, the resulting beverage displayed an increased bioactive profile. This enhancement was retained for a duration of 28 days in storage. Furthermore, its cellular antioxidant activity was also higher.

However, an intriguing observation arose regarding the source and nature of decreased lipid accumulation in 3T3-L1 cells when treated with both juices. It appears that phenolics, glucosinolates, vitamin C, and carotenoids, despite their variations, are not evidently responsible for their anti-obesogenic potential. The latter, carotenoids, are unlikely candidates, as they degrade. This suggests that other molecules present in one of the three plant matrices used as raw materials to produce the juice might be causing this effect, which should be further explored.

Overall, our findings suggest that wounding stress, as a pretreatment for carrots, could be a viable strategy for producing beverages with a heightened health-beneficial profile. This offers potential applications for the food and beverage industry interested in producing healthy products.

## Figures and Tables

**Table 1:** Proximate and dietary fiber analyses (%) of fresh juice simples.

Determinations	Samples ^i,ii,iii^	
	Control Juice (CJ, %)	Stressed Juice (SJ, %)
Moisture	90.6 ± 0.01	90.65 ± 0.02
Ash	0.44 ± 0.01	0.46 ± 0.001
Total carbohydrates	7.88 ± 0.01	7.94 ± 0.01
Total fat	0.14 ± 0.01 ^a^	<0.10 ^b^
Protein	0.94 ± 0.00 ^a^	0.95 ± 0.01 ^b^
Crude fiber	0.25 ± 0.00	0.38 ± 0.01
Total dietary fiber	0.82 ± 0.01	0.80 ± 0.01
Soluble dietary fiber	0.55 ± 0.01	0.54 ± 0.01
Insoluble dietary fiber	0.27 ± 0.00 ^a^	0.25 ± 0.01 ^b^

^i^ CJ, juice prepared with non-stressed carrots; SJ, juice prepared with stressed carrots. ^ii^ Values represent the mean of three replicates with their standard error. ^iii^ Different letters in the same row indicate statistically significant differences between juice samples by the LSD test (*p* < 0.05).

**Table 2:** Effects of storage (28 days at 4 °C) on the physicochemical parameters of juice samples.

Samples ^i^	Storage Time (Days)	Parameters ^ii,iii^			
		Acidity (g/L Citric Acid)	pH	Soluble Solids (°Bx)	Insoluble Solids (g/L)
Control Juice (CJ)	0	0.23 ± 0.001 ^b, A^	4.9 ± 0.03 ^a, A^	8.8 ± 0.03 ^a, A^	90.6 ± 0.06 ^a, A^
	14	0.24 ± 0.001 ^a, A^	4.8 ± 0.001 ^b, A^	8.7 ± 0.001 ^b, A^	89.5 ± 0.18 ^b, A^
	28	0.23 ± 0.001 ^b, A^	4.8 ± 0.001 ^b, A^	8.1 ± 0.001 ^c, A^	90.09 ± 0.01 ^a, A^
Stressed Juice (SJ)	0	0.23 ± 0.001 ^a, A^	4.8 ± 0.03 ^a, B^	8.7 ± 0.06 ^a, B^	91.2 ± 0.12 ^a, A^
	14	0.27 ± 0.001 ^c, B^	4.7 ± 0.001 ^b, B^	8.7 ± 0.03 ^a, A^	90.6 ± 0.44 ^a, b, A^
	28	0.25 ± 0.001 ^b, B^	4.7 ± 0.01 ^b, B^	8.4 ± 0.03 ^b, B^	90.9 ± 0.08 ^b, A^

^i^ CJ, juice prepared with non-stressed carrots; SJ, juice prepared with stressed carrots. ^ii^ Values represent the means of three replicates with their standard error. ^iii^ Different minor letters in the same column, within the same juice sample, indicate statistically significant differences over time, and different capital letters in the same column indicate statistically significant differences between juice samples in the same time point yielded by the LSD test (*p* < 0.05).

**Table 3:** Effects of storage time (28 days at 4 °C) on color CIE LAB values of juice samples.

Samples ^i^	Storage Time (Days)	Parameters ^ii,iii^				
		L*	a*	b*	Chroma	Hue
Control Juice (CJ)	0	7.43 ± 0.008 ^a, A^	26.77 ± 0.006 ^a, A^	12.57 ± 0.02 ^a, A^	29.57 ± 0.003 ^a, A^	25.16 ± 0.04 ^a, A^
	14	8.64 ± 0.017 ^b, A^	27.68 ± 0.008 ^b, A^	14.73 ± 0.006 ^b, A^	31.35 ± 0.01 ^b, A^	28.01 ± 0.007 ^b, A^
	28	14.01 ± 0.02 ^c, A^	31.19 ± 0.021 ^c, A^	24.02 ± 0.08 ^c, A^	39.36 ± 0.06 ^c, A^	37.59 ± 0.07 ^c, A^
Stressed Juice (SJ)	0	11.47 ± 0.01 ^a, B^	26.11 ± 0.001 ^a, A^	19.3 ± 0.02 ^a, B^	32.46 ± 0.01 ^a, B^	36.47 ± 0.03 ^a, B^
	14	17.24 ± 0.003 ^b, B^	15.14 ± 0.006 ^b, B^	7.99 ± 0.01 ^b, B^	17.12 ± 0.006 ^b, B^	27.80 ± 0.04 ^b, B^
	28	10.15 ± 0.04 ^c, B^	26.94 ± 0.05 ^c, B^	17.41 ± 0.07 ^c, B^	31.24 ± 0.08 ^c, B^	33.85 ± 0.06 ^c, B^

^i^ CJ, juice prepared with non-stressed carrots; SJ, juice prepared with stressed carrots; L*, lightness; a*, negative value indicates red color/positive value indicates green; b*, negative value indicates blue color/positive value indicates yellow. ^ii^ Values represent the mean of three replicates with their standard error. ^iii^ Different minor letters in the same column, within the same juice sample, indicate statistically significant differences over time, and different capital letters in the same column indicate statistically significant differences between juice samples in the same time point determined by the LSD test (*p* < 0.05).

## Data Availability

The data used to support the findings of this study can be made available by the corresponding author upon request.

## References

[1] Barquera S., Rivera J.A. (2020). Obesity in Mexico: Rapid Epidemiological Transition and Food Industry Interference in Health Policies. Lancet Diabetes Endocrinol..

[2] Williams D.J., Edwards D., Hamernig I., Jian L., James A.P., Johnson S.K., Tapsell L.C. (2013). Vegetables Containing Phytochemicals with Potential Anti-Obesity Properties: A Review. Food Res. Int..

[3] Sharma K.D., Karki S., Thakur N.S., Attri S. (2012). Chemical Composition, Functional Properties and Processing of Carrot—A Review. J. Food Sci. Technol..

[4] Sakhi A.K., BØhn S.K., Smeland S., Thoresen M., Smedshaug G.B., TausjØ J., Svilaas A., Karlsen A., Russnes K.M., Svilaas T. (2010). Postradiotherapy Plasma Lutein, α-Carotene, and β-Carotene Are Positively Associated with Survival in Patients with Head and Neck Squamous Cell Carcinoma. Nutr. Cancer.

[5] Sharma K.D., Stähler K., Smith B., Melton L. (2011). Antioxidant Capacity, Polyphenolics and Pigments of Broccoli-Cheese Powder Blends. J. Food Sci. Technol..

[6] Jaswir I., Noviendri D., Hasrini R.F., Octavianti F. (2011). Carotenoids: Sources, Medicinal Properties and Their Application in Food and Nutraceutical Industry. J. Med. Plants Res..

[7] Kasperczyk S., Dobrakowski M., Kasperczyk J., Ostałowska A., Zalejska-Fiolka J., Birkner E. (2014). Beta-Carotene Reduces Oxidative Stress, Improves Glutathione Metabolism and Modifies Antioxidant Defense Systems in Lead-Exposed Workers. Toxicol. Appl. Pharmacol..

[8] Zhang D., Hamauzu Y. (2004). Phenolic Compounds and Their Antioxidant Properties in Different Tissues of Carrots (*Daucus carota* L.). J. Food Agric. Environ..

[9] Santana-Gálvez J., Cisneros-Zevallos L., Jacobo-Velázquez D.A. (2017). Chlorogenic Acid: Recent Advances on Its Dual Role as a Food Additive and a Nutraceutical against Metabolic Syndrome. Molecules.

[10] Jacobo-Velázquez D.A., Cisneros-Zevallos L. (2023). Does Consumption of Tortured Fruit and Vegetables Improve Health, and Do They Taste Good?. ACS Food Sci. Technol..

[11] Cisneros-Zevallos L., Jacobo-Velázquez D.A. (2020). Controlled Abiotic Stresses Revisited: From Homeostasis through Hormesis to Extreme Stresses and the Impact on Nutraceuticals and Quality during Pre- and Postharvest Applications in Horticultural Crops. J. Agric. Food Chem..

[12] Jacobo-Velázquez D.A. (2023). Transformation of Carrots into Novel Food Ingredients and Innovative Healthy Foods. Appl. Food Res..

[13] World Health Organization (2015). Guideline: Sugars Intake for Adults and Children.

[14] Malik V.S., Hu F.B. (2022). The Role of Sugar-Sweetened Beverages in the Global Epidemics of Obesity and Chronic Diseases. Nat. Rev. Endocrinol..

[15] Talalay P., Fahey J.W., Holtzclaw W.D., Prestera T., Zhang Y. (1995). Chemoprotection against Cancer by Phase 2 Enzyme Induction. Toxicol. Lett..

[16] Santana-Gálvez J., Villela Castrejón J., Serna-Saldívar S.O., Jacobo-Velázquez D.A. (2020). Anticancer Potential of Dihydrocaffeic Acid: A Chlorogenic Acid Metabolite. CyTA-J. Food.

[17] Santana-Gálvez J., Santacruz A., Cisneros-Zevallos L., Jacobo-Velázquez D.A. (2019). Postharvest Wounding Stress in Horticultural Crops as a Tool for Designing Novel Functional Foods and Beverages with Enhanced Nutraceutical Content: Carrot Juice as a Case Study. J. Food Sci..

[18] (1994). Secretaría de Salud. Bienes y Servicios. Método Para la Cuenta de Bacterias Aerobias en Placa.

[19] (2014). Secretaría de Salud. Productos y Servicios. Métodos de Prueba Microbiológicos. Determinación de Microorganismos Indicadores. Determinación de Microorganismos Patógenos.

[20] (1994). Secretaría de Salud. Bienes y Servicios. Método Para La Cuenta de Mohos y Levaduras En Alimentos.

[21] (1986). Secretaría de Economía. Alimentos. Determinación de Humedad En Productos Alimenticios.

[22] (1980). Secretaría de Economía. Alimentos Determinación de Proteínas.

[23] (2004). NORMEX. Alimentos—Determinación de Extracto Etéreo.

[24] (1978). Secretaría de Economía. Determinación de Fibra Cruda En Alimentos.

[25] (2020). NORMEX. Alimentos. Determinación de Cenizas en Alimentos—Método de Prueba.

[26] (2008). NORMEX. Alimentos. Determinación de Fibra Dietética Fracción Insoluble y Fracción Soluble (Método Gravimétrico—Enzimático) en Alimentos—Método de Prueba.

[27] Villarreal-García D., Nair V., Cisneros-Zevallos L., Jacobo-Velázquez D.A. (2016). Plants as Biofactories: Postharvest Stress-Induced Accumulation of Phenolic Compounds and Glucosinolates in Broccoli Subjected to Wounding Stress and Exogenous Phytohormones. Front. Plant Sci..

[28] Gillespie K.M., Ainsworth E.A. (2007). Measurement of Reduced, Oxidized and Total Ascorbate Content in Plants. Nat. Protoc..

[29] Kumar P., Nagarajan A., Uchil P.D. (2018). Analysis of Cell Viability by the MTT Assay. Cold Spring Harb. Protoc..

[30] Cheenpracha S., Park E.-J., Rostama B., Pezzuto J.M., Chang L.C. (2010). Inhibition of Nitric Oxide (NO) Production in Lipopolysaccharide (LPS)-Activated Murine Macrophage RAW 264.7 Cells by the Norsesterterpene Peroxide, Epimuqubilin A. Mar. Drugs.

[31] Davies C.A., Rocks S.A., O’ Shaughnessy M.C., Perrett D., Winyard P.G., Winyard P.G., Willoughby D.A. (2003). Analysis of Nitrite and Nitrate in the Study of Inflammation. Inflammation Protocols.

[32] Tsikas D. (2007). Analysis of Nitrite and Nitrate in Biological Fluids by Assays Based on the Griess Reaction: Appraisal of the Griess Reaction in the l-Arginine/Nitric Oxide Area of Research. J. Chromatogr. B.

[33] Kellett M.E., Greenspan P., Pegg R.B. (2018). Modification of the Cellular Antioxidant Activity (CAA) Assay to Study Phenolic Antioxidants in a Caco-2 Cell Line. Food Chem..

[34] Zebisch K., Voigt V., Wabitsch M., Brandsch M. (2012). Protocol for Effective Differentiation of 3T3-L1 Cells to Adipocytes. Anal. Biochem..

[35] Popkin B.M., D’Anci K.E., Rosenberg I.H. (2010). Water, Hydration, and Health. Nutr. Rev..

[36] Lee S.R., Yi S.A., Nam K.H., Ryoo R., Lee J., Kim K.H. (2019). Pantheric Acids A–C from a Poisonous Mushroom, Amanita Pantherina, Promote Lipid Accumulation in Adipocytes. J. Nat. Prod..

[37] Olalude C.B., Oyedeji F.O., Adegboyega A.M. (2015). Physico-Chemical Analysis of Daucus Carota (Carrot) Juice for Possible Industrial Applications. IOSR J. Appl. Chem..

[38] Aderinola T.A., Abaire K.E. (2019). Quality Acceptability, Nutritional Composition and Antioxidant Properties of Carrot-Cucumber Juice. Beverages.

[39] Chuku E.C., Akani N.P. (2015). Determination of Proximate Composition and Microbial Contamination of Fresh Juice from Three Citrus Species. IIARD Int. J. Biol. Med. Res..

[40] Banigo E.B., Kiin-Kabari D.B., Owuno F. (2015). Physicochemical and Sensory Evaluation of Soy/Carrot Drinks Flavoured with Beetroot. Afr. J. Food Sci. Technol..

[41] Dima F., Istrati D., Garnai M., Serea V., Vizireanu C. (2015). Study on Obtaining Vegetables Juices with High Antioxidant Potential, Preserved by Ohmic Pasteurization. J. Agroaliment. Process. Technol..

[42] Braide W., Oranusi S.U., Otali C.C. (2012). Nutritional, Antinutritional, Minerals and Vitamin Compositions of Fourteen Branfs of Fruit Juice Sold in Onitsha Main Market. Res. Basic Appl. Sci..

[43] Yanaka A. (2017). Daily Intake of Sulforaphane-Rich Broccoli Sprouts Normalizes Bowel Habits in Healthy Human Subjects. FASEB J..

[44] Bokić J., Škrobot D., Tomić J., Šeregelj V., Abellán-Victorio Á., Moreno D.A., Ilić N. (2022). Broccoli Sprouts as a Novel Food Ingredient: Nutritional, Functional and Sensory Aspects of Sprouts Enriched Pasta. LWT.

[45] Popoola-Akinola O.O., Raji T.J., Olawoye B. (2022). Lignocellulose, Dietary Fibre, Inulin and Their Potential Application in Food. Heliyon.

[46] Niu L., Wu J., Liao X., Chen F., Wang Z., Zhao G., Hu X. (2008). Physicochemical Characteristics of Orange Juice Samples From Seven Cultivars. Agric. Sci. China.

[47] Romeo R., Bruno A.D., Piscopo A., Medina E., Ramírez E., Brenes M., Poiana M. (2020). Effects of Phenolic Enrichment on Vitamin C and Antioxidant Activity of Commercial Orange Juice. Braz. J. Food Technol..

[48] Salehi F. (2020). Physico-Chemical and Rheological Properties of Fruit and Vegetable Juices as Affected by High Pressure Homogenization: A Review. Int. J. Food Prop..

[49] Yu L.J., Rupasinghe H.P.V. (2012). Effect of Acidification on Quality and Shelf-Life of Carrot Juice. Can. J. Plant Sci..

[50] Aguiló-Aguayo I., Brunton N., Rai D.K., Balagueró E., Hossain M.B., Valverde J. (2014). Polyacetylene Levels in Carrot Juice, Effect of pH and Thermal Processing. Food Chem..

[51] Bharate S.S., Bharate S.B. (2014). Non-Enzymatic Browning in Citrus Juice: Chemical Markers, Their Detection and Ways to Improve Product Quality. J. Food Sci. Technol..

[52] Zhu D., Shen Y., Xu L., Cao X., Lv C., Li J. (2019). Browning and Flavor Changing of Cloudy Apple Juice during Accelerated Storage. IOP Conf. Ser. Mater. Sci. Eng..

[53] Munsch M.H., Simard R.E., Girard J.-M. (1983). Relationships in Colour and Carotene Content of Carrot Juices. Can. Inst. Food Sci. Technol. J..

[54] Leahu A., Damian C., Carpiuc N., Oroian M., Avramiuc M. (2013). Change in Colour and Physicochemical Quality of Carrot Juice Mixed with Other Fruits. J. Agroaliment. Process. Technol..

[55] Ferrario M., Guerrero S., Char C. (2017). Optimisation of Minimal Processing Variables to Preserve the Functional Quality and Colour of Carrot Juice by Means of the Response Surface Methodology. Int. J. Food Sci. Technol..

[56] Tiwari B.K., Muthukumarappan K., O’Donnell C.P., Cullen P.J. (2008). Colour Degradation and Quality Parameters of Sonicated Orange Juice Using Response Surface Methodology. LWT-Food Sci. Technol..

[57] Sirichan T., Kijpatanasilp I., Asadatorn N., Assatarakul K. (2022). Optimization of Ultrasound Extraction of Functional Compound from Makiang Seed by Response Surface Methodology and Antimicrobial Activity of Optimized Extract with Its Application in Orange Juice. Ultrason. Sonochem..

[58] Blanco Gomis D., Sánchez Núñez N., Dolores Gutiérrez Álvarez M. (2006). High Speed Liquid Chromatography for In-Process Control. J. Liq. Chromatogr. Relat. Technol..

[59] Ianni F., Barola C., Blasi F., Moretti S., Galarini R., Cossignani L. (2022). Investigation on Chlorogenic Acid Stability in Aqueous Solution after Microwave Treatment. Food Chem..

[60] Rodriguez-Concepcion M., Avalos J., Bonet M.L., Boronat A., Gomez-Gomez L., Hornero-Mendez D., Limon M.C., Meléndez-Martínez A.J., Olmedilla-Alonso B., Palou A. (2018). A Global Perspective on Carotenoids: Metabolism, Biotechnology, and Benefits for Nutrition and Health. Prog. Lipid Res..

[61] Alvarado-Ramírez M., Santana-Gálvez J., Santacruz A., Carranza-Montealvo L.D., Ortega-Hernández E., Tirado-Escobosa J., Cisneros-Zevallos L., Jacobo-Velázquez D.A. (2018). Using a Functional Carrot Powder Ingredient to Produce Sausages with High Levels of Nutraceuticals. J. Food Sci..

[62] Arvayo-Enríquez H., Mondaca-Fernández I., Gortárez-Moroyoqui P., López-Cervantes J., Rodríguez-Ramírez R. (2013). Carotenoids Extraction and Quantification: A Review. Anal. Methods.

[63] Chen H.E., Peng H.Y., Chen B.H. (1996). Stability of Carotenoids and Vitamin A during Storage of Carrot Juice. Food Chem..

[64] Bell T., Alamzad R., Graf B.A. (2016). Effect of pH on the Chemical Stability of Carotenoids in Juice. Proc. Nutr. Soc..

[65] Chanson-Rolle A., Braesco V., Chupin J., Bouillot L. (2016). Nutritional Composition of Orange Juice: A Comparative Study between French Commercial and Home-Made Juices. Food Nutr. Sci..

[66] Gong Y., Yu J.-Y., Qian P., Meng J., Zhang X.-J., Lu R.-R. (2015). Comparative Study of the Microbial Stability and Quality of Carrot Juice Treated by High-Pressure Processing Combined with Mild Temperature and Conventional Heat Treatment. J. Food Process Eng..

[67] Pérez-Balibrea S., Moreno D.A., García-Viguera C. (2010). Glucosinolates in Broccoli Sprouts (*Brassica oleracea* Var. *Italica*) as Conditioned by Sulphate Supply during Germination. J. Food Sci..

[68] Oliviero T., Verkerk R., Van Boekel M.A.J.S., Dekker M. (2014). Effect of Water Content and Temperature on Inactivation Kinetics of Myrosinase in Broccoli (*Brassica oleracea* Var. *Italica*). Food Chem..

[69] Burmeister W.P., Cottaz S., Rollin P., Vasella A., Henrissat B. (2000). High Resolution X-Ray Crystallography Shows That Ascorbate Is a Cofactor for Myrosinase and Substitutes for the Function of the Catalytic Base. J. Biol. Chem..

[70] Wang J., Barba F.J., Frandsen H.B., Sørensen S., Olsen K., Sørensen J.C., Orlien V. (2016). The Impact of High Pressure on Glucosinolate Profile and Myrosinase Activity in Seedlings from Brussels Sprouts. Innov. Food Sci. Emerg. Technol..

[71] Hanschen F.S., Lamy E., Schreiner M., Rohn S. (2014). Reactivity and Stability of Glucosinolates and Their Breakdown Products in Foods. Angew. Chem. Int. Ed..

[72] Jacobo-Velázquez D.a., Cisneros-Zevallos L. (2009). Correlations of Antioxidant Activity against Phenolic Content Revisited: A New Approach in Data Analysis for Food and Medicinal Plants. J. Food Sci..

[73] Li A.-N., Li S., Zhang Y.-J., Xu X.-R., Chen Y.-M., Li H.-B. (2014). Resources and Biological Activities of Natural Polyphenols. Nutrients.

[74] Zhang H., Liu R., Tsao R. (2016). Anthocyanin-Rich Phenolic Extracts of Purple Root Vegetables Inhibit pro-Inflammatory Cytokines Induced by H2O2 and Enhance Antioxidant Enzyme Activities in Caco-2 Cells. J. Funct. Foods.

[75] Ning W., Peng X., Ma L., Cui L., Lu X., Wang J., Tian J., Li X., Wang W., Zhang L. (2012). Enhanced Secondary Metabolites Production and Antioxidant Activity in Postharvest Lonicera Japonica Thunb. in Response to UV Radiation. Innov. Food Sci. Emerg. Technol..

[76] Villamil-Galindo E., Antunes-Ricardo M., Piagentini A.M., Jacobo-Velázquez D.A. (2022). Adding Value to Strawberry Agro-Industrial by-Products through Ultraviolet A-Induced Biofortification of Antioxidant and Anti-Inflammatory Phenolic Compounds. Front. Nutr..

[77] Zhang L., Virgous C., Si H. (2019). Synergistic Anti-Inflammatory Effects and Mechanisms of Combined Phytochemicals. J. Nutr. Biochem..

[78] Anzano A., Bonanomi G., Mazzoleni S., Lanzotti V. (2022). Plant Metabolomics in Biotic and Abiotic Stress: A Critical Overview. Phytochem. Rev..

[79] Naveed M., Hejazi V., Abbas M., Kamboh A.A., Khan G.J., Shumzaid M., Ahmad F., Babazadeh D., FangFang X., Modarresi-Ghazani F. (2018). Chlorogenic Acid (CGA): A Pharmacological Review and Call for Further Research. Biomed. Pharmacother..

[80] Hsu C.-L., Yen G.-C. (2007). Effects of Flavonoids and Phenolic Acids on the Inhibition of Adipogenesis in 3T3-L1 Adipocytes. J. Agric. Food Chem..

[81] Sudhakar M., Sasikumar S.J., Silambanan S., Natarajan D., Ramakrishnan R., Nair A.J., Kiran M.S. (2020). Chlorogenic Acid Promotes Development of Brown Adipocyte-like Phenotype in 3T3-L1 Adipocytes. J. Funct. Foods.

